# A Rare Case of Guillain-Barré Syndrome Post-gastrojejunostomy and Literature Review

**DOI:** 10.7759/cureus.35889

**Published:** 2023-03-08

**Authors:** Hamzh D Jaber, Hamzah M Magableh, Mohamad D Jaber, Ahmad M Magableh, Faisal H Ghazal, Ayman Z Azzam, Tarek Amin

**Affiliations:** 1 College of Medicine, Alfaisal University, Riyadh, SAU; 2 Radiology Department, Southend University Hospital, Southend-on-Sea, GBR; 3 General Surgery Department, Faculty of Medicine, Alexandria University, Alexandria, EGY; 4 Surgical Oncology Department, Oncology Center, King Faisal Specialist Hospital and Research Center, Riyadh, SAU

**Keywords:** postoperative complication, surgery, literature review, case report, gastrojejunostomy, guillain-barré syndrome

## Abstract

Guillain-Barré syndrome (GBS) is a rare immune-mediated neuropathy causing destruction of the peripheral nervous system, with molecular mimicry playing a major role in its pathophysiology. Despite its rarity, it is considered the most common cause of acute flaccid neuromuscular paralysis in the United States. Although diagnosing GBS depends on the clinical presentation of the patient, cerebrospinal fluid sampling, nerve conduction studies, electromyography, magnetic resonance imaging, and ganglioside antibody screening can be used to confirm the diagnosis and rule out other differentials. Here, we report a rare case of GBS as a postoperative complication after a successful gastrojejunostomy to excise an adenocarcinoma in the second part of the duodenum. Such a complication is rare and not fully understood yet.

## Introduction

Guillain-Barré syndrome (GBS) is a rare clinically diagnosed autoimmune disease, leading to acute peripheral neurological destruction. The three main types of the disease are acute inflammatory demyelinating polyradiculoneuropathy (AIDP), Miller Fisher syndrome (MFS), and acute motor axonal neuropathy (AMAN) [1,2]. Each subtype has a unique pathophysiology and clinical presentation, yet all subtypes are caused by an autoimmune attack on various parts of the peripheral nervous system.

The majority of GBS cases are due to prior stressful events leading to autoimmune activation, with 70% of patients complaining of infections one to six weeks prior to the presentation of GBS. Other events can also cause GBS such as vaccination, trauma, or surgeries [1]. Malignancy is considered a major risk factor for developing GBS secondary to abnormal immune reactions [3,4]. A retrospective study suggested that surgeries account for an attributable risk of 4.1 GBS cases per 100,000 surgeries [5]. Clinically, patients present with symmetrical ascending and flaccid weakness and numbness. Diagnosis can be done via clinical assessment, yet further investigations are needed to confirm the diagnosis and rule out other differentials.

In the majority of cases, the disease can be well controlled, with intravenous immunoglobulins (IVIG) as the main treatment. Plasmapheresis can also be considered in acutely deteriorating cases [1].

Here, we present the case of a patient at our tertiary center who developed GBS post-surgical excision of gastrointestinal cancer. Reporting our experience will help enrich the clinical literature and provide insights into such a rare complication and its possible management options.

## Case presentation

A 53-year-old male presented to the hospital’s outdoor clinic with a five-month history of progressive food intolerance, nausea, vomiting multiple times a day, and abdominal distention, all of which are considered signs of gastrointestinal obstruction. His symptoms were getting worse with time. During the last two months, the patient had an unintentional weight loss of 29 kg which was associated with progressive loss of appetite. Apart from these, the patient did not have any other symptoms and no signs of infections.

Total blood count, liver functions tests, renal functions tests, electrolyte profile, and coagulation profile were all within the normal range. Based on laboratory values, history of presenting illness, and clinical presentation, gastrointestinal cancer was our top differential. Further investigations were done to confirm the diagnosis.

**Figure 1:**
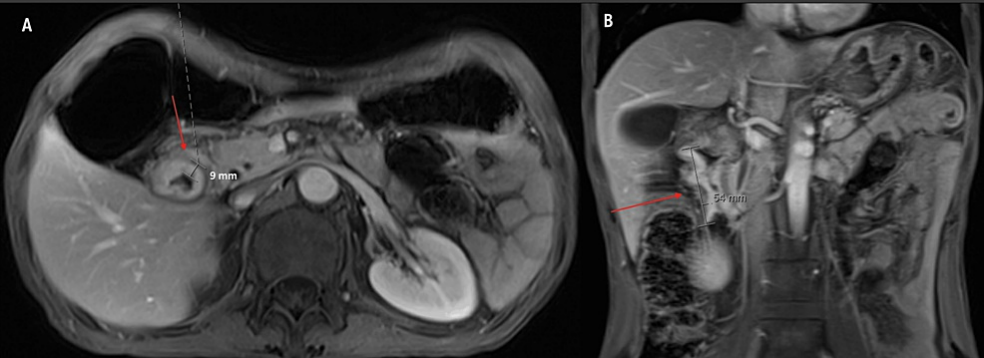
T1-weighted MRI with contrast. Cross-sectional T1-weighted MRI (A): Circumferential annular constricting duodenal wall thickening along the second duodenal segment, with the lesion measuring 9 mm in thickness. Coronal T1-weighted MRI (B): The lesion measures 54 mm in length and appears inseparable from the ampulla of Vater and the pancreatic head. MRI = magnetic resonance imaging

**Figure 2:**
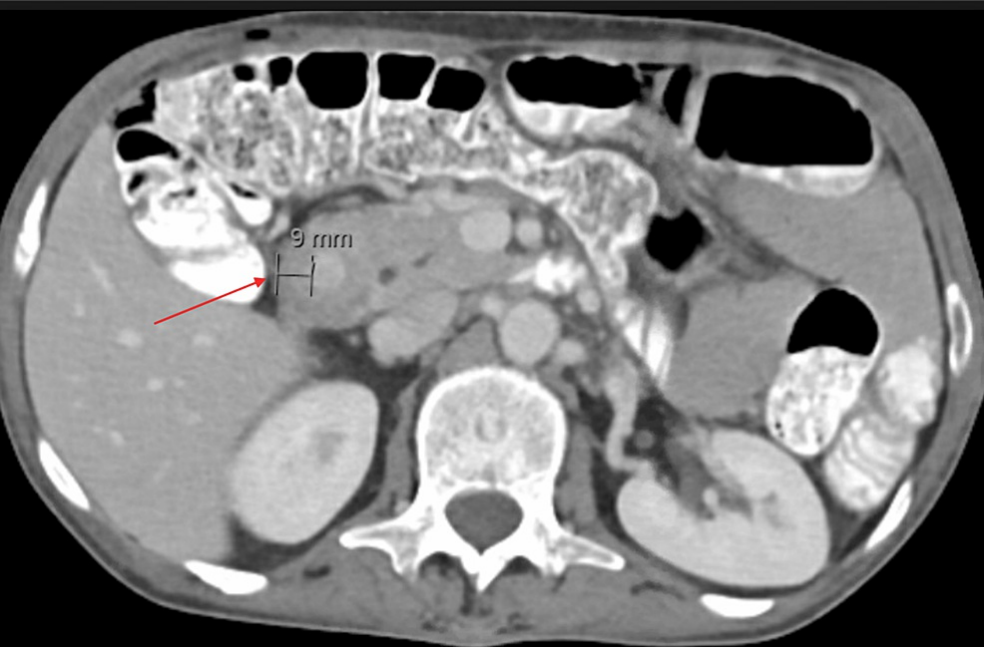
CT with contrast. A 9 mm thickening of the second part of the duodenum. CT = computerized tomography

**Figure 3:**
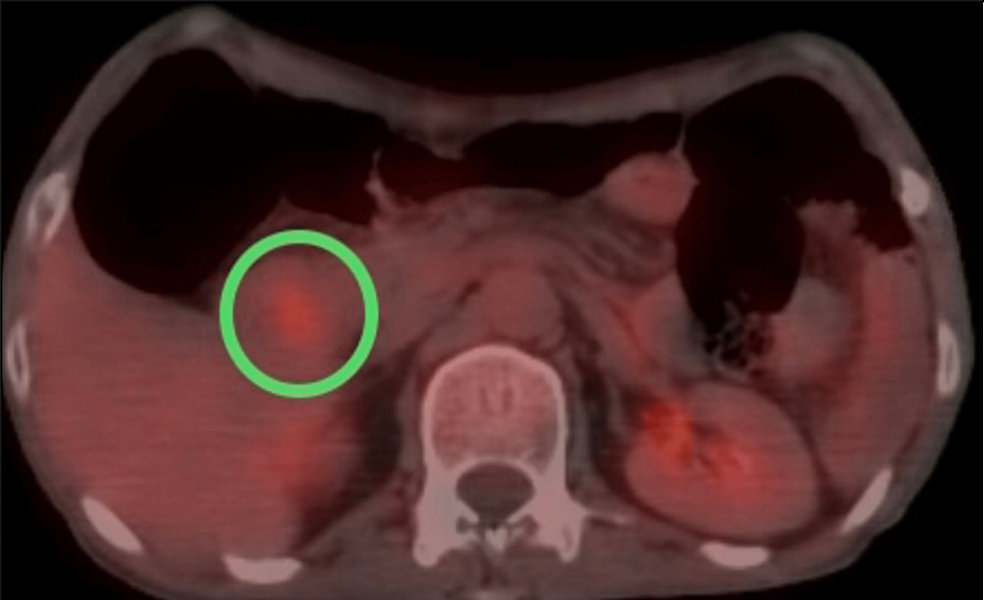
PET-CT. Cross-sectional PET-CT showing an increased FDG-avid activity in the second part of the duodenum. PET-CT = positron emission tomography-computerized tomography; FDG = fluorodeoxyglucose

Magnetic resonance imaging (MRI) showed a circumferential constricting mass in the second part of the duodenum, measuring 9 mm in thickness and 54 mm in length, as shown in Figure 1. Computerized tomography (CT) scan showed a mass in the second part of the duodenum with associated gastric outlet obstruction, as shown in Figure 2. Esophagogastroduodenoscopy (EGD) reported irregular thickening of the second part of the duodenum, and a biopsy confirmed the presence of poorly differentiated adenocarcinoma. Positron emission tomography-computerized tomography (PET-CT) scans showed an increased fluorodeoxyglucose (FDG) uptake in the second part of the duodenum, with an absence of metastasis (Figure 3). Tumor cells were positive for AE1/AE3.

The patient was scheduled for surgical treatment followed by chemotherapy. He underwent a laparoscopic gastrojejunostomy under general anesthesia. The procedure was successful with an estimated blood loss of 70 mL. The patient was followed up after the surgery with an absence of complications including infections or leakage.

After one week of the surgery, the patient started to complain of numbness and weakness in his upper and lower limbs, which was progressive and ascending over time, with difficulty in walking as well. Electromyography (EMG) showed predominantly axonal length-dependent sensorimotor polyneuropathy with demyelination, and cerebrospinal fluid (CSF) showed elevated protein levels with normal white blood cell count (cytoalbuminologic dissociation). Apart from that, CSF pressure, appearance, glucose, and Gram stain were normal.

Based on the clinical presentation and the EMG findings, the patient was diagnosed with GBS AIDP and started on IVIG 0.4 g/kg for five days. Due to the patient’s newly diagnosed disease, chemotherapy was delayed till further assessments. After five days, the patient did not show improvement but there was no progression or respiratory failure.

## Discussion

GBS is considered the most common cause of acute flaccid paralysis, with an annual global incidence of 1-2 per 100,000. It affects males more than females, with incidence increasing with age [4]. The majority of patients suffer from an infection prior to GBS, most commonly due to *Campylobacter jejuni*, Zika virus, and the severe acute respiratory syndrome coronavirus 2 [6]. It is important to note that in the majority of cases, the mechanism of the autoimmune attack is due to molecular mimicry between the invading agent and the peripheral nervous system [6,7]. Infections are not the only cause of the disease as any prior stressful event such as surgeries and trauma can lead to disease development [2].

Although the pathophysiology of GBS is not well understood, many studies have helped scientists to understand and appreciate the unique clinical-pathological phenotypes of the disease, resulting in better overall management and prevention planning for affected patients [2]. Newer antibody-mediated model studies have suggested that the driving factor behind the AMAN subtype of GBS is an antibody-mediated attack on axolemma driven by molecular mimicry, as in a majority of GBS cases, the body’s immune system creates antibodies against lipooligosaccharides (LOS) presented on the surface of the invading organism [2]. Anti-LOS antibodies can bind to structurally identical glycans on nerve gangliosides [2]. Anti-ganglioside antibodies are of IgG1 and IgG3 subclasses, binding mainly to monosialotetrahexosylganglioside (GM1) and N-acetylgalactosaminyl (GD1a) gangliosides. Reported experiments were able to understand the cascade of events leading to disruption of the anatomical and physiological structure and integrity, as it starts with antibody binding, fixing complement, and recruiting macrophages, which leads to depositing membrane attack complexes [2].

Our patient was diagnosed with the AIDP subtype of the disease. Although the exact pathophysiology of AIDP is less understood in contrast to other subtypes, it starts with a wide range of immune stimulants including surgeries causing an acute inflammatory reaction that develops into AIDP [2]. To date, there is no specific set of known antibodies responsible for the disease [2]. Some studies have shown T and B cells are responsible for the immune response to gliomedin, contactin, transient axonal glycoprotein-1 (Tag-1), membrane-organizing extension spike protein (Moesin), neurofascin, and compact myelin proteins, such as P0, P2, and PMP22 [2,8,9]. A study reported antibodies against moesin triggered by cytomegalovirus (CMV) infections, indicating a possible association with GBS secondary to CMV; however, it is important to note that the result of this study was not replicable [10]. An interesting new study has shown that anti-complex antibodies which can attack a complex of multiple lipid and glycolipid components instead of attacking only a single molecule can play a major role in the development of GBS [2,10]. There are ongoing studies to discover these anti-complex antibodies and develop future study plans [11,12].

The challenging aspect of diagnosing GBS is the fact that there are multiple subtypes of the disease, each with its unique clinical features and investigation findings. The most useful investigation to differentiate between different subtypes is EMG [4]. The initial diagnostic approach should be based on the clinical presentation of the disease. Typically, in patients presenting with sensory loss in the legs and possibly the arm, the disease rapidly progresses to muscular weakness with decreased to absent reflexes [4]. If there is an absence of central nervous system involvement or other possible causes, the diagnosis of GBS can be made based on the clinical presentation. Many patients can have an atypical presentation such as severe and diffuse or local pain accompanied by cranial nerve dysfunction and many other possible variants and symptoms [2,4]. The presence of atypical presentation does not rule out GBS, and further confirmatory investigations are needed.

CSF examinations are expected to show an elevated CSF protein with normal CSF cell count [2,4]. Normal CSF protein levels do not rule out GBS as protein levels remain normal in 10-30% of patients in the second week of the disease’s presentation and normal in 30-50% during the first week [4]. EMG studies are a sufficient tool to confirm the diagnosis. For AIDP, demyelination is considered a hallmark of the disease, which was the situation in our case [7]. EMG can be done at least once in the third week of symptom presentation in case previous studies showed normal results, as in the third week 90% of patients will show an abnormal result, leading to a more accurate diagnosis [7].

A retrospective study reviewing patients who developed GBS within six weeks after the surgery confirmed the presence of a strong statistical correlation between GBS and gastrointestinal surgeries, general anesthesia, and malignancy [3]. In the study, 42.1% of patients had active malignancy, with 21% suffering a gastrointestinal malignancy, making it the most common malignancy in the study [3]. It is important to note that only 21% of GBS patients in the study developed a post-surgical infection prior to GBS, which confirms that infections are not the only cause of developing the disease [3]. The study reported that 95% of patients underwent general anesthesia, because of which a comparison between the effect of different types of anesthesia on the development of GBS could not be made [3]. Patients with post-surgical GBS had a longer hospital stay with a poorer prognosis compared to patients who did not undergo surgery [3].

Our patient was initially diagnosed with duodenal adenocarcinoma, for which he underwent laparoscopic gastrojejunostomy under general anesthesia. The patient did not develop any infections after the surgery and did not need a blood transfusion. He started developing typical GBS presentation one week after his surgery, such as numbness and weakness in his upper and lower limbs, which was progressive and ascending over time, resulting in difficulty walking. The diagnosis was confirmed by CSF analysis which showed an elevated protein count and normal cell count. EMG revealed generalized predominantly axonal length-dependent sensorimotor polyneuropathy with demyelination. Based on the clinical presentation and the investigations, GBS was confirmed, with AIDP being the most likely subtype based on the EMG findings.

The typical treatment plan for GBS includes regular IVIG infusion in addition to plasmapheresis in case of failed treatment. Our patient was given IVIG 0.4 g/kg for five days without improvement. This might be because postoperative GBS patients have an overall poorer response to treatment [13].

The patient’s cascade of events goes along with AIDP, as surgical procedures are considered a major triggering factor leading to autoimmune stimulation and activation [2]. Considering this case is a rare combination of GBS risk factors that are not well understood, we believe reporting it will help in understanding GBS as a postoperative complication and achieve a better understanding of how surgical procedures might lead to it.

## Conclusions

GBS is a rare autoimmune disorder affecting the peripheral nervous system. GBS is caused by prior autoimmunity stimulants including infections, surgeries, and trauma in some documented causes. There are multiple subtypes of GBS, including AMAN, AIDP, and MFS. Diagnosis is done by the clinical presentation, yet many patients can present with atypical symptoms, indicating further testing such as EMG, CSF analysis, and MRI. Treatment comprises IVIG and plasmapheresis.
